# Dissolving Microneedles for Rapid and Painless Local Anesthesia

**DOI:** 10.3390/pharmaceutics12040366

**Published:** 2020-04-17

**Authors:** Byeong-Min Lee, Chisong Lee, Shayan Fakhraei Lahiji, Ui-Won Jung, Gehoon Chung, Hyungil Jung

**Affiliations:** 1 Department of Oral Physiology and Program in Neurobiology, School of Dentistry, Seoul National University, Seoul 08826, Korea; 2 Dental Research Institute, Seoul National University, Seoul 08826, Korea; 3 Department of Biotechnology, Building 123, Yonsei University, 50 Yonsei-ro, Seodaemun-gu, Seoul 03722, Korea; 4 Department of Periodontology, Research Institute for Periodontal Regeneration, Yonsei University College of Dentistry, Seoul 03722, Korea; 5 Juvic Biotech, Inc., No. 208, Digital-ro 272, Guro-gu, Seoul 08389, Korea

**Keywords:** dissolving microneedles, lidocaine, local anesthesia, centrifugal lithography, carboxymethyl cellulose

## Abstract

Microneedles are emerging drug delivery methods for painless treatment. The current study tested dissolving microneedles containing lidocaine (Li-DMN) for use in local anesthesia. An Li-DMN patch was fabricated by centrifugal lithography with carboxymethyl cellulose as a structural polymer and assessed for physical properties by optical microscopy and a fracture force analyzer. The biocompatibility was evaluated by a histology section in vitro and by ear thickness in vivo. The efficacy of the Li-DMN patch was assessed by electrophysiological recordings in primary cultured sensory neurons in vitro and a von Frey test on rats’ hind paws in vivo. The physical properties of the microneedle showed enough rigidity for transdermal penetration. The maximal capacity of lidocaine-HCl in the Li-DMN patch was 331.20 ± 6.30 µg. The cytotoxicity of the dissolving microneedle to neuronal cells was negligible under an effective dose of lidocaine for 18 h. Electrophysiological recordings verified the inhibitory effect of the voltage-gated sodium channel current by the Li-DMN patch in vitro. A skin reaction to the edema test and histologic analysis of the rats’ ears after application of the Li-DMN patch were negligible. Also, the application of the Li-DMN patch reduced the nocifensive behavior of the rats almost immediately. In conclusion, the dissolving microneedle patch with carboxymethyl cellulose is a promising candidate method for the painless delivery of lidocaine-HCl.

## 1. Introduction

Local anesthetic drugs block the transmission of nerve impulses by the inhibition of voltage-gated sodium channels (VGSCs) [1,2]. However, injection of local anesthetics causes various discomfort, ranging from a brief sting to trypanophobia and denial of medical operations. Topical creams or sprays containing local anesthetics have been developed as a means to avoid needle injections [3,4,5,6]. These needleless topical slow agents gained popularity for their convenience in the relevant application. However, the use of topical anesthetics is limited due to their slow onset and short duration. The onset time of topical anesthetics is slow because local anesthetic agents need to be released and diffuse into skin before reaching peripheral nerves [7,8]. The minute amount of an active ingredient in a topical application requires frequent re-applications during medical treatment. Thus, while they could be applied by medical professionals or even by patients conveniently, the anesthetic effect is slow and incomplete.

To improve the onset time, the pretreatment of microneedles, micro-scale needle structures, before the application of topical local anesthetics has been proposed. Penetration of the stratum corneum, the outermost skin barrier, by microneedles was presumed to assist the permeation of topical local anesthetics at the epidermal layer [9]. This approach required a separate application of the microneedles and topical anesthetics. The inconvenience of a two-step application was improved by coating anesthetic agents on the surface of the microneedles made of metal, glass or non-degradable polymer materials [10,11,12]. However, the drug-coated microneedle (CMN) was not successful in the clinical environment because of the limited amount of drug-coated materials and the low biocompatibility and biohazardous waste of the microneedle materials [10,13,14]. Therefore, CMNs were soon replaced by dissolving microneedles (DMNs), which encapsulated drugs within a homogenized biodegradable polymer [14,15,16]. DMNs penetrate the stratum corneum and dissolve into the interstitial fluid, thereby releasing the encapsulated drugs at the epidermal layer [14,17,18,19]. As the capacity for local anesthetics was low in the biodegradable polymer used for the DMNs, the attachment of a large number of DMNs or a reservoir of anesthetics to DMN patches was required to release a sufficient amount [19]. The manufacturing process and the clinical application was difficult, and the slow release of the anesthetic ingredient from the reservoir was unfavorable for rapid and effective local anesthetics.

Recently, a novel DMN platform using centrifugal lithography, which enables the encapsulation of drugs with high concentrations, was developed [15,18]. The current study aims to test the newly developed DMN platform as a convenient, rapid and highly effective local anesthetic system without extra treatment or reservoirs. The DMN patch containing lidocaine-HCl (Li-DMN patch) was fabricated using centrifugal lithography with carboxymethyl cellulose (CMC) as a structural polymer for the viscosity, bio-degradability and biocompatibility [20,21].

The CMC-based Li-DMN patch was analyzed for morphological and mechanical properties required for the insertion of DMNs through the skin. The cell viability and anesthetic efficacy of the Li-DMN patch were tested using neuron cells in vitro. Furthermore, the safety and efficacy of the Li-DMN patch were confirmed and compared with the topical anesthetics for the rapid and effective anesthetics in the rodent model in vivo.

## 2. Materials and Methods

### 2.1. Fabrication of Lidocaine-HCl-Loaded DMN Patches

Carboxymethyl cellulose (CMC, 90 kDa, low-viscosity, Sigma-Aldrich, St. Louis, MO, USA) was selected as the biodegradable polymer for the Li-DMNs. The polymer–drug mixture solution was prepared by mixing 14% (*w*/*v*) of CMC with the dissolved lidocaine-HCl monohydrate (Sigma-Aldrich) in distilled water (DW) and homogenized using a vacuum mixer (ARV-310, Thinky, Tokyo, Japan). The prepared mixture solution was dispensed as an array of 25 droplets (5 × 5) onto the thin CMC film using a robotic dispenser (SHOTmini 100S, Musashi Engineering Inc., Tokyo, Japan). Droplets were shaped and solidified into DMNs using centrifugal lithography [15]. DMN patches without lidocaine-HCl (blank DMN patches) were fabricated by the same method for the control group experiments.

### 2.2. Physical Properties of Lidocaine-HCl-Loaded DMN Patches

Images of DMN patches were obtained using a stereomicroscope (M165FC, Leica Camera AG, Wetzlar, Germany) and a digital microscope camera (DFC450C, Leica Camera AG). The fracture force of the DMNs was analyzed using a force analyzer (Z0.5TN, Zwick Roell Inc., Ulm, Germany). A single DMN was placed on the test stage and a metal probe was moved downward at a continuous speed of 3.6 mm/min. After the probe reached the tip of the DMN, the axial force when the fracture of the DMN occurred was recorded as the fracture force.

### 2.3. Quantitative Analysis of Lidocaine-HCl Content in DMN Patches

A single Li-DMN patch with 5 × 5 arrays was dissolved in 1 mL of DW and analyzed using a high-performance liquid chromatography system (HPLC, Waters 600S, Waters, Milford, MA, USA). A C18 column (150 × 4.6 mm I.D., YMC-Triart C18, YMC Co., Ltd., Kyoto, Japan) was used as a reverse phase. A stock solution of lidocaine-HCl was serially diluted (0~500 µg/mL, R^2^ ≥ 0.99) with distilled water to prepare the calibration curve. The mobile phase A with acetonitrile and phase B with a 1% (*v*/*v*) solution of trifluoroacetic acid in DW were used for the experiment. The phase ratio A–B (30:70) was isocratic, and the flow rate was 1.0 mL/min. The detection wavelength of lidocaine-HCl was 254 nm.

### 2.4. Evaluation of Cytotoxicity of DMN Patches in Vitro

The cytotoxicity of the blank and Li-DNM patches was evaluated by a colorimetric cell viability assay sensitive to lactate dehydrogenase (LDH) activity, called CCK-8 assay (Dojindo Molecular Technologies, Inc., Kumamoto, Japan), of an F11 (ECACC 08062601) cell line that derived from rat embryonic dorsal root ganglion cells [22]. Briefly, F11 cells were plated in 96 well plates (8 × 10^3^ cells per well) and incubated for 24 h before assay. Lidocaine powder, DMN patches with or without lidocaine, and a lidocaine cartridge used in dental clinics were diluted in serum-free Dulbecco’s Modified Eagle Medium (DMEM) media with an equal concentration of lidocaine, and F11 cells were treated on each group for 2, 3, 6, 12 and 18 h. After cells were washed with Hanks balanced salt solution (HBSS; Welgene, Daegu, Korea), one-hundred microliters of CCK-8 solution was added to each well and was incubated for an additional 1 h at 37 °C. The optical density (OD) of each well at 450 nm was measured by a microplate reader (Epoch2, Biotek, Seoul, Korea). The cell viability (relative to control) is expressed as the ratio value of (OD_test_−OD_blank_)/(OD_control_−OD_blank_), where OD_test_ is the optical density of the cells exposed to the test solutions, OD_control_ is the optical density of lidocaine-untreated cells and OD_blank_ is the optical density of the wells without cells.

### 2.5. Animals

All surgical and experimental procedures were reviewed and approved by the Institutional Animal Care and Use Committee at Seoul National University (SNU-170308-8-1 from Mar. 08, 2017 to Mar. 26, 2019 and SNU-170807-8-1 from Sep. 11, 2017 to Sep. 10, 2019). Animal treatments were performed in accordance with the Guidelines of the International Association for the Study of Pain. Male Sprague-Dawley rats (approximately weighing 200~270 g at the time of the experiment) were housed at a temperature of 23 ± 2 °C with a 12-h light–dark cycle and fed food and water *ad libitum*. The animals were allowed to habituate in the housing facilities for 1 week before the experiments, and efforts were made to limit distress to the animals.

### 2.6. Assessment of Tissue Reaction of DMN Patch on Rat Ear

Adult rats (8~9 weeks old) were anesthetized with pentobarbital sodium (50 mg/kg i.p.) and the DMN patch was applied on the inner surface of an ear. The skin response to the DMN patch was assessed by measuring the increase in the ear thickness at the edge of the ear at 10, 30, 60 and 120 min [23], after which rats were sacrificed for a histologic examination. The ears of each rat were removed and fixed with 10% formalin in phosphate-buffered saline (PBS). Sections of the ear tissue were stained with hematoxylin/eosin for microscopic evaluation.

### 2.7. Whole-Cell Patch Clamp Recording

Trigeminal ganglion (TG) neurons from 8~9 weeks old adult rats (200–270 g) were prepared as previously described [24]. Briefly, TG neurons prepared in Hanks’ Balanced Salt Solution (HBSS) at 4 °C were incubated in 2 mL HBSS containing 0.17% trypsin (Invitrogen, Carlsbad, CA, USA) at 37 °C for 35 min. The cells were washed with DMEM and triturated with a flame-polished Pasteur pipette, and were subsequently centrifuged (1500 RPM, 10 min). After seeding on poly-d-Lysin (Sigma-Aldrich)-coated glass coverslips, the cells were maintained in neurobasal media (Gibco, Carlsbad, CA, USA) at 37 °C and 5% CO_2_ condition.

The VGSC current was recorded by whole-cell patch clamp recordings using electrodes (4–6 MΩ) pulled from borosilicate glass on a vertical micropipette puller (PC-100, Narishige, Tokyo, Japan). Voltage clamp experiments were performed using a HEKA EPC10 USB amplifier (HEKA Electronik, Lambrecht, Germany). Signals were filtered at 1 kHz and sampled at 3 kHz. Electrical recordings were performed at room temperature and data were analyzed by FitMaster software (HEKA Electronik, Lambrecht, Germany).

The extracellular solution containing 140 mM of NaCl, 1 mM of MgCl_2_, 5 mM of KCl, 10 mM of HEPES, 10 mM of D-glucose and 2 mM of EGTA (ethylene glycol-bis(β-aminoethyl ether)-*N*,*N*,*N*′,*N*′-tetraacetic acid) was adjusted to pH 7.4 with NaOH, with the osmotic concentration of 290~300 mOsm. Pipettes were filled with an intracellular solution containing 135 mM of CsCl, 5 mM of MgCl_2_, 10 mM of HEPES, 5 mM of EGTA, 10 mM of d-glucose and 2 mM of Mg-ATP, adjusted to pH 7.4 with CsOH, with the osmotic concentration of 290~300 mOsm. Cells were continuously perfused with the extracellular solution using a gravity-fed perfusion system. The recording was started at least 5 min after obtaining the whole-cell configuration

### 2.8. Behavior Test for Measuring Mechanical Pain Threshold

Sprague-Dawley rats (between 8 and 9 weeks) were habituated and the hind paw withdrawal threshold was measured with von Frey hair filaments by the “up-and-down method” [25]. The animals were acclimated for at least 30 min in a customized cage before each experiment. The Li-DMN or blank DMN patches were applied on the right plantar skin of rats for 1 min and the von Frey test was conducted in a blind manner immediately after the detachment of the DMN patches, and repeated after 10 and 30 min, and 1, 2 and 3 h. As a control, a topical 9.6% lidocaine cream (Hanmi Pharm Inc., Seoul, Korea) containing an identical amount (3.45 mg) of lidocaine as the Li-DMN patch was applied on the middle area of the right plantar, where a von Frey filament tip was applied for 1 min and a von Frey test was performed following the same protocol with the patch applied group.

### 2.9. Statistical Analysis

All values were expressed as the mean ± SEM and were compared by two-tailed t-tests for a comparison of two groups and one-way ANOVA for several groups after the normality test. The statistical analysis was performed with Prism 8.0. (GraphPad Software, La Jolla, CA, USA).

## 3. Results

### 3.1. Physical Properties of DMN Patches

**Figure 1:**
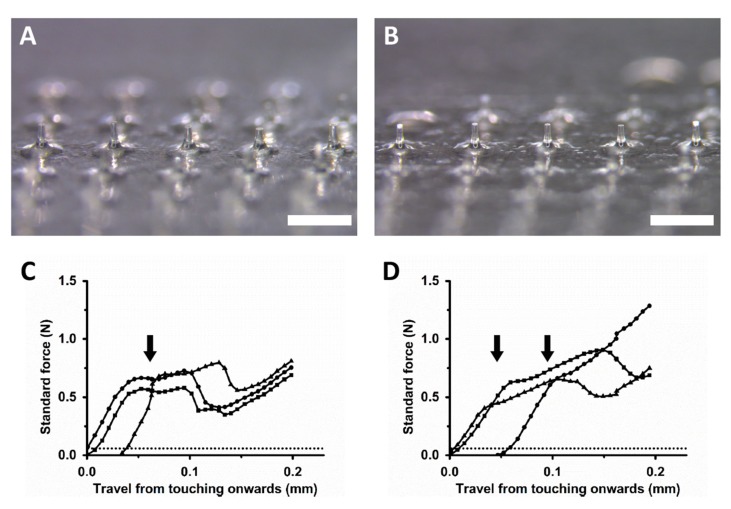
Morphological observation and fracture force analysis of dissolving microneedles encapsulated with lidocaine-HCl. (**A**,**B**) Microscopic images of dissolving microneedles without lidocaine-HCl for the control group and with lidocaine-HCl for the experimental group, respectively. The scale bar represents 1000 µm. (**C**,**D**) Fracture force analysis results of dissolving microneedles without and with lidocaine-HCl, respectively. Arrow and dashed lines indicate the moment when the fracture occurs and the minimum strength required for skin penetration (0.058 N), respectively.

The 5 × 5 mm Li-DMN patches each containing 25 DMNs were fabricated. The size and shape were determined by considering the typical local anesthetic application area for dermatological or dental surgery. Each Li-DMN patch contained 331.20 ± 6.30 µg of lidocaine-HCl. The average length, base diameter and a tip diameter of the uniform array of DMNs were 369.62 ± 11.64 μm, 688.60 ± 16.56 μm and 65.86 ± 2.33 μm, respectively, for DMN without lidocaine-HCl and 376.34 ± 8.39 μm, 671.05 ± 11.40 μm and 63.17 ± 2.33 μm, respectively, for DMN with lidocaine-HCl (Figure 1A,B). The fracture forces of the single DMN measured with a force analyzer were 0.65 ± 0.06 N without lidocaine-HCl and 0.65 ± 0.01 N with lidocaine-HCl (*n* = 3, arrows on Figure 1C,D).

### 3.2. Cytotoxicity of DMN Patch Solutions

**Figure 2:**
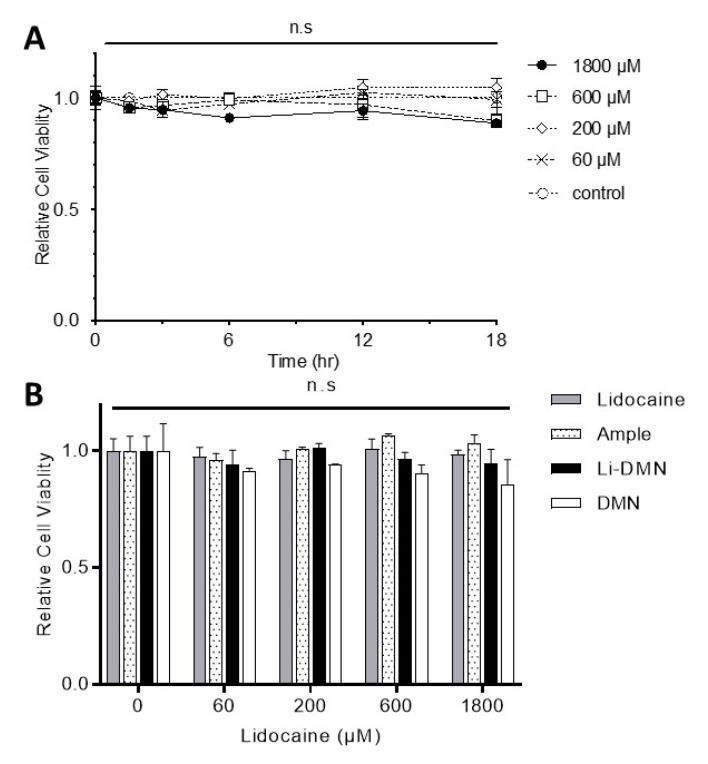
The effects of lidocaine containing solutions on F11 cell viability that was evaluated with CCK-8 assay. (**A**) Different concentrations (60, 200, 600, 1800 μM) of lidocaine-HCl containing Li-DMN patch dissolved media was exposed to cells and was viability assessed after 2, 3, 6, 12 and 18 h of incubation at 37 °C and they show no significant effects on the viability of F11 cells. (**B**) Different types of lidocaine contained solution (lidocaine powder dissolved, dissolving microneedles containing lidocaine (Li-DMN) dissolved, and lidocaine ample solution for clinic usage) was treated on F11 cells and they also did not show significant cytotoxicity up to 18 h of treatment.

The cytotoxic effect of the Li-DMN patches was evaluated with a colorimetric cell viability assay, sensitive to LDH activity. Since the IC_50_ of lidocaine-HCl to the VGSC is about 60 μM, the DMN solutions with 60, 200, 600 and 1800 μM of lidocaine-HCl were tested on the F11 cells, immortalized murine dorsal root ganglion cells, for 1, 2, 6, 12 and 18 h. DMN patches showed negligible cytotoxicity at all concentrations of lidocaine-HCl tested up to 18 h (Figure 2A). The CCK-8 cytotoxic assay results were compared with a commercially available 2% lidocaine-HCl cartridge solution and lidocaine-HCl solution by the dilution of the powder (>99% purity). DMN patches without lidocaine-HCl were used as a control group. Testing of the commercial lidocaine-HCl cartridge or freshly diluted 200 μM lidocaine-HCl for up to 18 h showed no significant cytotoxic effect in the F11 cells (Figure 2B).

### 3.3. Inhibition of VGSC Current by Lidocaine from DMN Patches

**Figure 3:**
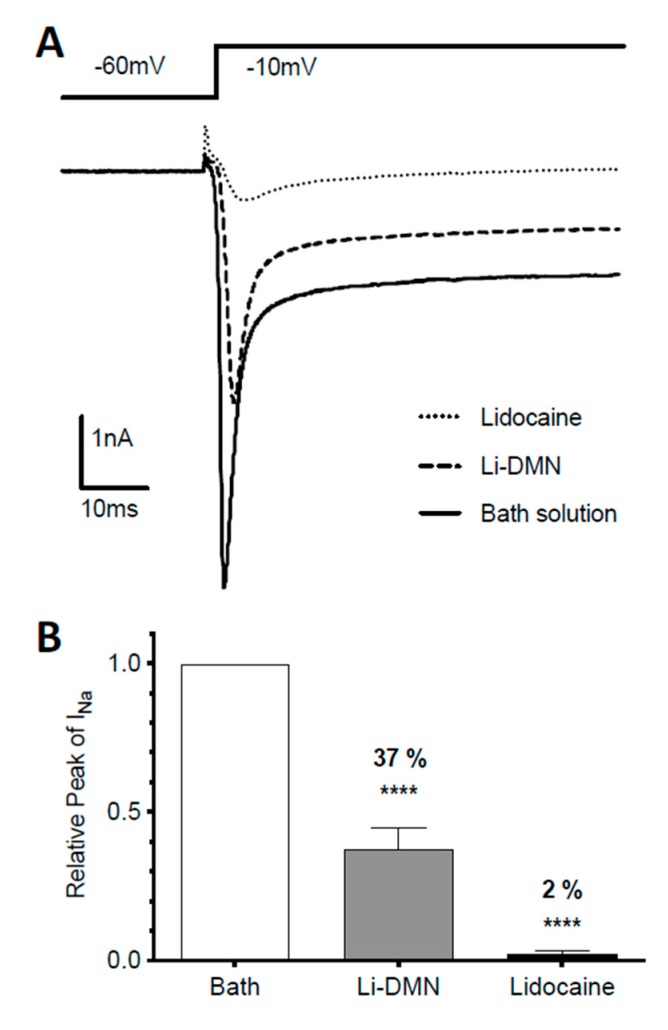
Sodium current recorded with whole-cell patch clamping. (**A**) Representative trace of inward sodium current that before and after exposure to the 200 μM lidocaine-HCl solution or the Li-DMN patch dissolved solution contains an equal concentration of lidocaine. Inward sodium current was induced by step depolarization to −10 mv from holding potential −60 mV for 30 ms. (**B**) Quantitative comparison of relative sodium current that were measured before and after treatment of the Li-DMN patch solution or lidocaine solution. Data are shown as mean ± SEM. A paired t-test was used for analysis (**** *p* < 0.0001).

The ability of the Li-DMN patch to inhibit the VGSC was evaluated by whole-cell patch clamp recordings of the primary cultured TG neurons. Typical VGSC currents were evoked by experimental depolarization of the membrane potential to −10 mV from the holding potential of −60 mV (Figure 3A). The amplitudes of the VGSC currents were significantly inhibited by the Li-DMN (*n* = 7, 62.5% ± 7.1%, Figure 3A,B). However, the inhibition was less than that by the same concentration (200 μM) of the lidocaine-HCl solution (98.1% ± 1.3% inhibition).

### 3.4. Tissue Response to DMN Patch Application

**Figure 4:**
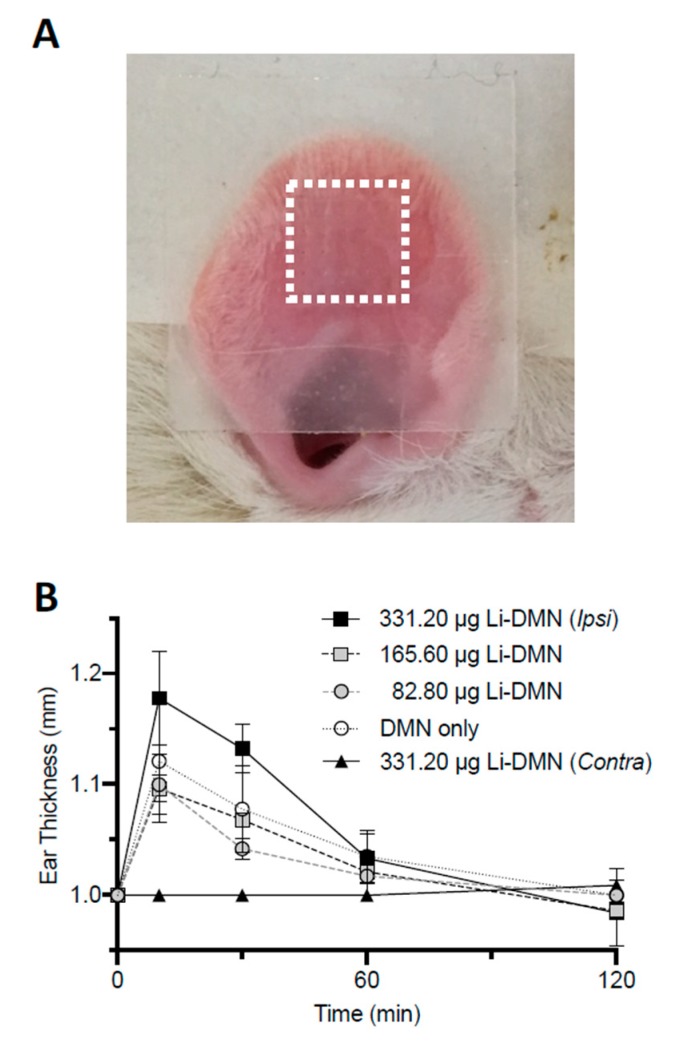
Ear thickness change after dissolving microneedle (DMN) patch application on the ear of the rat. (**A**) Image of the applicated Li-DMN patch on the ear of the rat. The dashed square indicates the location of the DMN array. (**B**) Measurement of ear thickness at 10, 30, 60 and 120 min after application of the DMNs that contain different doses of lidocaine-HCl. Comparison of ear thickness change according to time between the ipsilateral and contralateral side of the DMN patch application that contains the maximal dose (331.20 μg), a half-maximal dose (165.60 μg) and a quarter-maximal dose (82.80 μg) of lidocaine-HCl and the blank DMN patch without lidocaine-HCl and the contralateral ear as controls, respectively. One-way ANOVA and a paired t-test were used for statistical analysis.

The potential tissue damage after the Li-DMN patch application was evaluated by observation of the ear thickness, as previously suggested [26]. The thickness of the ear edge of 8~9 weeks old adult rats was measured after the application of the Li-DMN patch containing three different doses of lidocaine-HCl: 331.20 ± 6.30 µg (maximum dose), 1/2 and 1/4 of the maximum. The Li-DMN patch was applied at the hairless inner surface of the ears, as shown in Figure 4A with the dashed square. Although slight indentations corresponding to the 5 × 5 array shape of the Li-DMN patch were recognized, no signs of tissue damage, such as redness or bleeding, were observed on the surface of the ear. The ear thickness was slightly increased 10 min after the Li-DMN patch application with the max, 1/2 and 1/4 concentrations, but within statistical insignificance, and it was gradually recovered within 60 min (Figure 4B). Furthermore, the Li-DMN patches with different lidocaine-HCl doses and blank DMN patch showed a similar trend in ear thickness increment and recovery.

**Figure 5:**
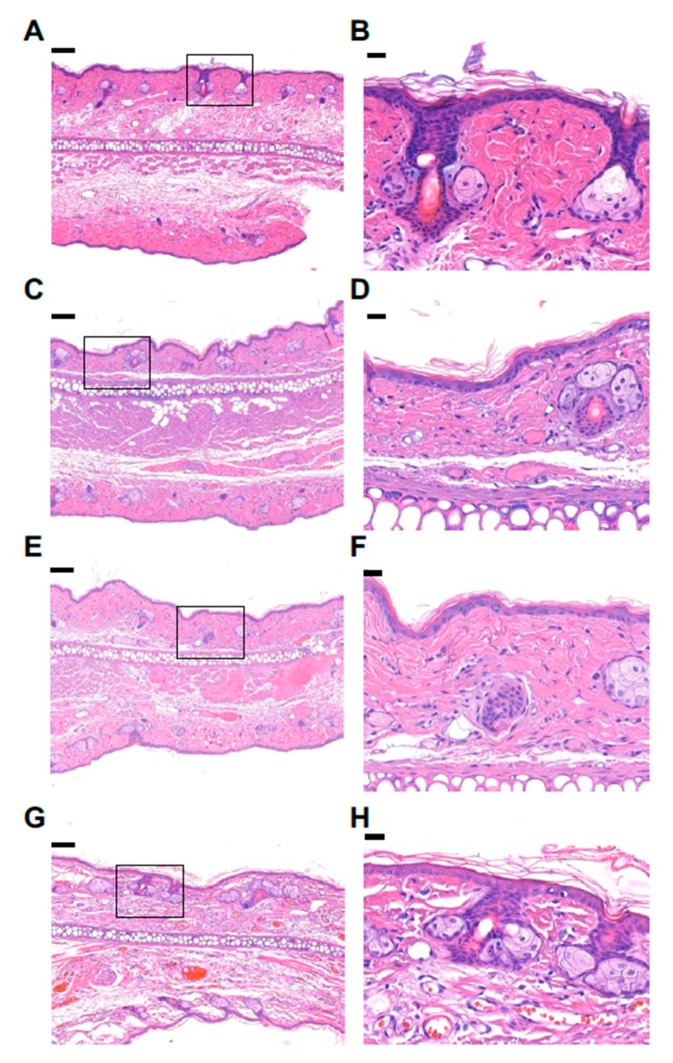
Representative Haemotoxylin & Eosin stained histology sections of the ear after DMN patch application. The left column shows ear sections after 120 min of DMN patch application. The right column represents the patch application site (the rectangular sub-sections shown in the left column) (**A**,**B**) DMN patch containing maximal dose (331.20 μg) of lidocaine-HCl (**C**,**D**) DMN patch containing a half-maximal dose (165.60 μg) of lidocaine-HCl (**E**,**F**) DMN patch containing a quarter maximal dose (82.80 μg) of lidocaine-HCl (**G**,**H**). Blank DMN patch without lidocaine-HCl. Red arrows indicate DMN insertion sites. Scale bar indicates 100 μm and 20 μm in the left and the right column, respectively.

Similar findings were identified in the histological observation. The Li-DMN patches, regardless of the concentration of lidocaine-HCl, did not induce any atypical changes in anatomic structures, such as hair follicles, glands and cartilage, or any signs of inflammation including increased epithelial thickness and migration of inflammatory cells or edema (Figure 5). The skin channel from the DMN were not detected in our histological sections. However, the proper application of the DMNs was checked by that the microneedles on the DMNs were completely dissolved after 1 min of application. The enlarged histological images of each group (Figure 5, right column) showed normal vascularity and fiber density at the patch application site. These results indicate that the Li-DMN patch has a diminutive negative effect on rats’ skin. As no tissue response was observed in the histological observation, the Li-DMN patch containing a maximum dose of lidocaine-HCl was selected for the following in vivo anesthetic efficacy test.

### 3.5. Li-DMN Patches Diminished Nociception Of Animals

**Figure 6:**
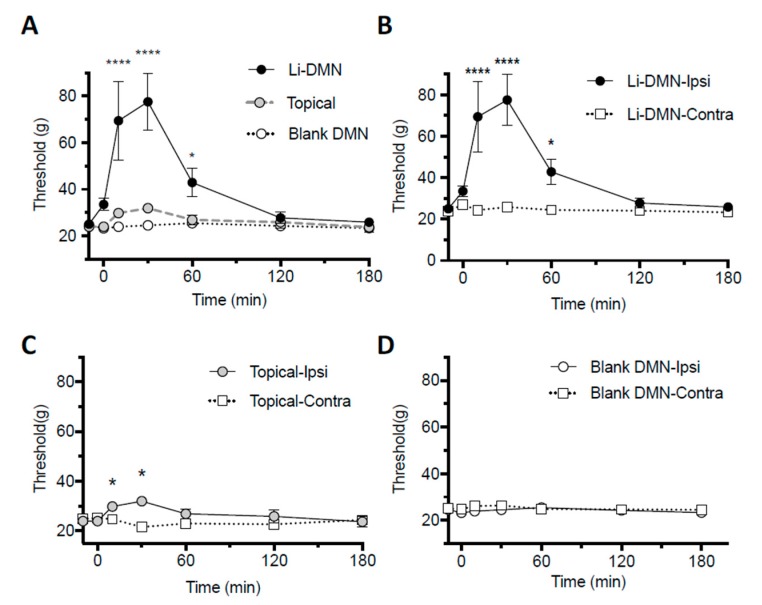
Behavioral test to evaluate the anesthetic effect of the DMN patches on the mechanical pain threshold. Von Frey test was used to estimate the mechanical pain threshold by the up-and-down method by calculating the 50% paw withdrawal threshold. (**A**) Anesthetic efficacy of the Li-DMN patch on rat paw compared with a blank patch that did not contain lidocaine-HCl and the commercial topical lidocaine agent. Comparing the mechanical pain threshold according to time between ipsilateral and contralateral sides after (**B**) the maximal dose of Li-DMN patch, (**C**) the topical lidocaine cream and (**D**) the blank DMN patch application, respectively. Data were shown as mean ± SEM (*n* = 6). Two-way ANOVA and paired t-test were performed. * *p* < 0.05, **** *p* < 0.0001.

The Li-DMN patch applied on rats’ hind paws increased the paw withdrawal threshold significantly compared to the commercial topical lidocaine agent or control DMN patch that contained no anesthetic agent (ANOVA, *p* < 0.0001, Figure 6A). The paw withdrawal threshold was increased from 24.6 ± 0.5 g before the DMN patch application (data shown before 0 min in Figure 6) to 69.5 ± 16.9 g, 77.6 ± 12.3 g and 42.9 ± 6.0 g after 10, 30 and 60 min of the DMN patch application, respectively (two-tailed *t*-test, *p* = 0.0034, 0.0005 and 0.047, respectively).

The comparison of the paw withdrawal threshold between the ipsi- and contralateral sides after the Li-DMN application showed a significant difference (ANOVA with multiple comparisons, *p* < 0.0001) (Figure 6B). Although the topical lidocaine cream showed a significantly different paw withdrawal threshold between the ipsi- and contralateral sides, the effect was less than that of the Li-DMN (ANOVA with multiple comparisons, *p* < 0.05, Figure 6C). The blank DMN patch with no lidocaine showed no difference in the paw withdrawal threshold between the ipsi- and contralateral sides (Figure 6D).

## 4. Discussion

In this study, the CMC-based Li-DMN patch fabricated through centrifugal lithography was tested for anesthetic efficiency and biocompatibility in vitro and in vivo. The rigidity of the Li-DMN patch and the amount of lidocaine contained in the Li-DMN patch was sufficient for clinical use. The lidocaine-HCl released from the Li-DMN patches inhibited the VGSC current in rat trigeminal ganglion neurons in vitro and the nocifensive behavior of rats in vivo. The onset was fast and the anesthetic efficacy at the early time point was higher than the commercially available topical anesthetic agents.

The physical properties of the Li-DMN patch were comparable to the previously reported DMN patch [18]. No significant differences in shape, size and fracture resistance in the DMN patch were caused by encapsulating lidocaine-HCl. The mechanical strengths were considered to be sufficient for skin penetration regardless of drug encapsulation [18]. The maximum amount of lidocaine-HCl contained in the DMN patch was similar to the dose required for clinical use as previously reported [19]. These results suggested that the CMC-based Li-DMN patch could be an effective means of painless local anesthesia.

No significant cytotoxicity nor inflammatory signs were detected in the DMN with the amount of lidocaine-HCl tested in this study. As the biological half-life of lidocaine inside the human body is approximately between 2 and 2.5 h [27], the absence of cellular toxicity up to 18 h of the Li-DMN treatment with the maximum dose tested is considered sufficient for clinical application. The ear thickness test revealed swelling of the ear after 10 min of the DMN patch, regardless of the lidocaine concentration. The reason for the temporary increase in ear thickness is not clear. Although the penetration by the DMN is limited to the outermost layer of skin, a slight increase in local blood flow could be induced. Nonetheless, the swelling was resolved to the normal thickness shortly after, and the histological examination did not indicate any sign of inflammation. These results suggested that the toxic effect of the DMN was minimal and no additional toxicity could be expected than conventional lidocaine.

An electrophysiological analysis of the primary cultured neuron under the Li-DMN solution revealed a reduced peak amplitude of the voltage-gated sodium current. Moreover, while lidocaine released from the Li-DMN was expected to reduce sodium current [28], the inhibition was less than that by the lidocaine-HCl solution of the same concentration. The diffusion of lidocaine into the cytosol of neurons might be impeded by the chemical reaction between the lidocaine-HCl and the structural polymer CMC in the DMN. The binding site of the VGSC and lidocaine is on the cytosol side of the cell membrane, meaning lidocaine has to make entry into the cell to take effect [29]. Lidocaine is a highly lipophilic molecule that easily diffuses across the cell membrane, but it becomes hydrophilic when its weak base amine group accepts a hydrogen ion (*pK*_a_ = 7.9). The structural polymer CMC in the DMN contains negatively charged carboxylate residues that might charge lidocaine positively, thereby reducing the active concentration of lidocaine in the cytosol. Nevertheless, these results demonstrate that the Li-DMN solution inhibited the VGSC current in vitro.

The reduced nocifensive behavior observed in the von Frey filament test suggested the anesthetic efficacy of the Li-DMN in vivo. Interestingly, the onset was almost immediate. The paw withdrawal threshold started to increase immediately after the application of the Li-DMN patch, showing a maximal anesthetic effect between 10 and 30 min after application. Moreover, while the fast onset could be clarified by careful further studies, it might result from the structural benefit of the DMN, which penetrates through the epidermis and delivers drugs from the subcutaneous level. Interestingly, topical lidocaine, even when the same amount of lidocaine was treated, increased the paw withdrawal threshold to a lesser extent than the Li-DMN. The pain threshold immediately after the Li-DMN patch application was higher than the highest value measured after the topical lidocaine application. In addition, the effect of the Li-DMN patch was substantially maintained for 60 min, longer than the topical anesthetics. These results suggested that the Li-DMN could be preferred over topical lidocaine for its rapid onset and higher efficacy.

In conclusion, the current study suggests the CMC-based Li-DMN patch fabricated through centrifugal lithography as a novel local anesthesia system. The Li-DMN patch contained a sufficient amount of lidocaine, eliminating needs for a reservoir or over-sized preparation. The physical properties were similar to the previous microneedles, showing rigidity for skin penetration. No hazardous effect was observed in the cytotoxic analysis in vitro and the tissue response test in vivo. An electrophysiological observation of neurons revealed the inhibition of the voltage-gated sodium channel current by the aqueous solution of the Li-DMN, although CMC seemed to hinder the effect of lidocaine in part. Moreover, behavioral analysis of the rats demonstrated reduced nocifensive behavior after the application of the Li-DMN patch. Interestingly, the effect was noticeably more excessive and quicker than the topical application of lidocaine. Taken together, these results suggest the Li-DMN as a carefully balanced drug delivery method that provides minimal invasion and discomfort, with an additional fast onset. In addition, further studies including proper sterilization and storage stability analysis would contribute to the successful distribution and application of the Li-DMN patch. In conclusion, the Li-DMN could serve as a novel painless local anesthetic method that overcomes the limitations of the conventional lidocaine injection and topical cream in the various medical fields.
